# Design of Infertility Monitoring System: Minimum Data Set Approach

**DOI:** 10.25122/jml-2018-0071

**Published:** 2019

**Authors:** Maryam Zahmatkeshan, Mojtaba Farjam, Niloofar Mohammadzadeh, Tayebeh Noori, Zahra Karbasi, Zahra Mahmoudvand, Majid Naghdi, Reza Safdari

**Affiliations:** 1. Health Information Management Department, School of Allied Medical Sciences, Tehran University of Medical Sciences, Tehran, Iran; 2. Noncommunicable Diseases Research Center, School of Medicine, Fasa University of Medical Sciences, Fasa, Iran; 3. Department of Anatomical Sciences, School of Medicine, Fasa University of Medical Science, Fasa, Iran

**Keywords:** Data elements, Infertility, Minimum data set, Monitoring System

## Abstract

Reproductive health is vital for human and infertility is also one of the most important challenges in the reproductive system. Infertility is one of the most common chronic health disorders, regardless of age. The Minimum Data Set (MDS) helps to manage infertility by monitoring and evaluating infertility interventions based on collecting data.

The development of MDS is an essential objective in order to implement an infertility monitoring system for the creation of standardized and effective data management through the provision of comprehensive and identical data elements for infertility.

This is a descriptive cross-sectional study conducted in 2017. The data has been collected from infertility clinics in the world, as well as WHO, CDC, ASRM, and ESHRE reports.

In order to decide on data elements, the Delphi technique was used using a questionnaire that contained data elements which were distributed among 12 experts including one reproductive endocrinology and infertility fellow, six obstetrician-gynecologists, two reproductive biologists, two urologists and one community medicine specialist using the 5 point Likert scale. The questionnaire was divided into two categories: managerial and clinical, each with 4 sections, and 60 and 940 data elements, respectively.

MDS is an essential tool for evaluating the infertility process. Using this tool will provide an opportunity to develop a set of quality care criteria that can be used to ensure the quality of infertility care.

## Introduction

One of the key issues is the global emphasis on public health policies and programs to increase the population and provide affordable, safe and well-planned and effective services for families [1]. According to statistics in 2010, 48.5 million couples were infertile in the world [2], and almost 10% of the world’s population suffers from infertility [3]. In a survey of married women conducted by the Centers for Disease Control and Prevention (CDC) in 2015 in the United States, 1.5 million women (6%) were infertile, and the number of women who have used infertility services so far has reached 7.4 million [4,5].

Infertility is one of the six maternal diseases/consequences which has been neglected in developing countries [6]. Infertility has many side effects and is one of the causes of instability in the lives of couples [3, 4]. Infertility or its treatment can cause mental stress, anxiety, and depression [4]. Clinical depression in infertile women is similar to that of women with heart disease or cancer [3].

Therefore, in order to fully understand the effects of infertility and measure progress in reducing this problem and to cope with the growth management of data and information, attention to the collection, analysis, interpretation, and use of infertility data are of great importance [7]. Meanwhile, the most important step in managing information is to collect accurate and complete data and Minimum Data Set (MDS) is one of the most effective tools for collecting emerging data which provide accurate access to health information and as a set of information elements in health information systems, improves the use of high-quality data and it is very useful for planning, developing, monitoring, managing, and evaluating performances [4]. MDSs, by providing standard data with the same definitions, provide national and international comparisons [8] and as a basis for disease information management, improves the quality of care and disease control [9–12].

This is a descriptive cross-sectional study conducted in 2017. Information resources, including related articles, reports and forms related to infertility clinics and texts were searched using the following keywords: minimum data set, MDS, core data, infertility, infertility form, IVF registry, ART registry, surveillance system, monitoring system in the PubMed database, Scopus, Cochrane and WHO, CDC and Google scholar websites from 1978 to 2017. Collected data, according to the standards set of American National Standard Institute (ANSI), previous articles and references book were classified into managerial and clinical categories [17, 18, 24-26] and a questionnaire was created based on the 5-point Likert scale with 5 answer categories: strongly agree = 5, agree = 4, no idea = 3, disagree = 2, strongly disagree = 1. At the end of each part, an empty row titled “Other Items” was considered for the addition of important data elements by experts. Content validity of the questionnaire was evaluated by 12 relevant experts from the University of Medical Sciences of Fasa, including one reproductive endocrinology and infertility fellow, six obstetrician-gynecologists, two reproductive biologists, two urologists and one community medicine specialist. Experts were asked to express their agreement with each of the elements based on the 5-point Likert scale. The acceptance criterion for the data elements in the final MDS was the level of agreement of the experts with a level of agreement of over 75% (score of 3.75 up to 5). Eventually, the final data elements of the MDS were obtained. The test-retest method was also used to ensure the reliability of the questionnaire. Thus, within 10 days, the experts were again asked to fill out the questionnaire for the second time. Spearman’s rank correlation coefficient was 85% for the reliability of the questionnaire.

The purpose of this research is to develop the MDS required for a national infertility monitoring system in order to accelerate and facilitate the prevention, diagnosis, and follow-up of infertility.

This study is a research project as a part of the Maryam zahmatkeshan Ph.D. thesis approved by Tehran University of Medical Sciences. Also, the ethics committee of the university approved this study (IR.TUMS.SPH.REC.1396.4142). Verbal informed consent was obtained from all subjects before the study.

### Statistical analysis

SPSS 16 software was used to analyze the data.

## Results

Of the 12 experts, 75% were female, and 25% were male. Table 1 shows the demographic information and characteristics of the participating practitioners.

**Table 1:** Demographic characteristics of participants in the decision using the Delphi technique.

Participants	Number	Gender	Age group	Education	Academic field	Experience (years)
Specialist of obstetrics and gynecology	7	Female: 7 Male: 0	30-35: 3 35-40: 2 >40 :2	Fellowship of reproductive endocrinology and infertility: 1 Specialist: 6	reproductive endocrinology and infertility, obstetrics and gynecology	>5 : 2
reproductive biologists	2	Female: 1 Male: 1	30-35: 0 35-40: 1 >40 : 1	PhD: 2	reproductive biology	<5:0 >5:2
urologists	2	Female: 0 Male: 2	30-35: 1 35-40: 0 >40 : 1	Specialist: 2	urology	<5:0 >5:2
community medicine	1	Female: 1 Male: 0	30-35: 0 35-40: 1 >40 : 0	Specialist: 2	community medicine	<5:1 >5:0

MDS was divided into two categories of managerial and clinical, each with 4 sections. The total number of final data elements in the managerial groups was 60 and 940 in the clinical groups. Table 2 shows the accepted data elements for managerial (section a) and clinical (section b) groups.

**Table 2:** Managerial and clinical data category for the Minimum Data Set for infertility

a) Managerial Data							
Data sections	Strongly Agree (%)	Agree (%)	No idea (%)	Disagree (%)	Strongly Disagree (%)	Average rates	Final number of data elements
Demographic Data	78.9	12.3	5.3	1.3	0	4.6	38
Insurance information	83.3	4.2	11.7	0.8	0	4.7	10
Primary Care Provider (PCP	77.4	3.6	10.7	7.1	0	4.5	7
Signature items	80	6.7	13.3	0	0	4.7	5
Total	-	-	-	-	-	-	60
**b) Clinical Data**							
**Data sections**	**Strongly Agree (%)**	**Agree (%)**	**No idea (%)**	**Disagree (%)**	**Strongly Disagree (%)**	**Average rates**	**Final number of data elements**
Menstrual history	81.4	17	1.6	0	0	4.8	26
Sexual Issues	84	14.3	1.7	0	0	4.8	25
Previous reviews	71.9	19.4	8.2	0.3	0	4.5	97
Previous treatment	81	15.1	3.9	0	0	4.7	57
Previous surgical procedures	83.3	14.8	1	0.5	0	4.8	32
IVF / ICSI cycles / or previous frozen embryos transfer	83.3	15.9	0.8	0	0	4.8	11
Medical history	71.6	20.8	7.2	0.4	0	4.5	191
The history of medicine	71.7	20.6	7.8	0	0	4.4	30
Social Issues	80.1	15.2	4	0.7	0	4.7	23
Family History	70.1	19.8	9.8	0.2	0	4.4	107
Pregnancy history	72.1	21.6	6.3	0	0	4.5	32
Causes of infertility	80.1	14.7	5.1	0	0	4.8	13
Andrology tests in men	72.6	22.6	4.9	0	0	4.5	24
Immunological examination in women	67.9	19	11.9	1.2	0	4.3	7
Features of the cycle	83.3	13.2	3.1	0.3	0	4.8	53
Interventional measures and methods: Ovules, fertilization and embryos, protocols, and the type of drugs used.	81.4	13.8	3.5	1.2	0	4.7	87
Treatment results: Pregnancy and childbirth	83	11.8	4.4	0.8	0	4.8	72
Complications	80.6	15.7	3.7	0	0	4.7	9
Donor information	75.2	14.2	7	0.6	0	4.5	44
Total	-	-	-	-	-	-	940

Examples of the managerial and clinical data elements that were accepted as the final set (over 75%) are listed in Tables 3 and 4.

**Table 3:** Examples of managerial data elements for an MDS for the Infertility Monitoring System

Part	Data elements
Demographic data	Clinical trial identifier, clinic name, patient’s name, date of birth (age), place of birth, contact information, age at the start of the treatment, race, ethnicity, marital status, duration of marriage, other
Insurance information	Insurance company name, patient relationship to insured (self, spouse, child, other), original patient insurance, insurer, address, insurance number, expiration date, other.
Primary Care Provider (PCP)	Name, medical system number, specialty, address, phone number, e-mail.
Signature items	Signature (Physician- Nursing- Reproductive Care Center- Patient), translator needed, date, other

**Table 4:** Examples of clinical data elements for an MDS Infertility Monitoring System

Part			Data elements
	**Causes of infertility**		Tubular factor (missing, obstructed or fallopian tubes in a freshly inoculated cycle), ovulation impairment (defective ovulation release) in the freshly non-hereditary cycle, infertility due to reduced ovarian reserve, endometriosis, uterine factors (uterine abnormalities), menopause, ovarian failure, unknown factors, various factors related to women, factors related to both men and women, male factors (azoospermia, oligospermia, teratozoospermia), other factors.
**Medical history**	**Sexual History**	**Menstrual history**	Age of first period, date of the last period, features and cycle length, any pre-menstrual symptoms (PMS) (bloating, breast tenderness, mood change), bleeding or spotting between periods, pattern of the menstrual cycle (regular/irregular/spotting before period, light flow/heavy flow, bleeding between cycles, no period), menstrual days, changes in the menstruation over the past two years, painful menstruation, severity of cramps, medications for reduce pain, Last Monthly period/ LMP(last menstrual period)/Previous Menstrual Period, early menopause, postmenopausal bleeding, other.
		**Sexual History**	discomfort with intercourse, dyspareunia (painful intercourse), use of lubricants, discharge, number (repetition) of intercourse per week/month, intercourse for ovulation, douche before or immediately after intercourse, postcoital bleeding, leaving bed after intercourse, problems with initiating or completing intercourse,plan intercourse for a specific time of cycle, ejaculation problem, erection problems, spermatic ejaculation into the bladder, secretion or penile pain, recent changes in sexual stimulation, Undescended testicles, scrotal or testicular pain, testicular damage requiring hospitalization, the average number of ejaculations per week, Age of first of shaving regularly, sexual satisfaction, sexual relation status, follow-up due to infection or sexually transmitted diseases (Chlamydia, Gonorrhea, Herpes, genital warts, Syphilis, AIDS, hepatitis), other.
	**previous examinations**		infection/ irritation of pelvic organs, yeast infection, bladder infection (UTI), pelvic inflammatory disease (PID), polycystic ovary syndrome (PCOS), Endometrial Polyp(s),Ovarian Cyst(s), Endometriosis, Strep B, Herpes, Syphilis, HPV, genital warts, Gonorrhea, Chlamydia, Crabs, Trichomonas, Vaginitis, bacterial vaginosis, past semen analyses, prostate infection, penile infection, impotence, varicocele, gastroesophageal reflux disease, the time length to get pregnant now, the length of time when the contraceptive method is not used, Prior Testing: Post Coital Test (PCT), Endometrial Biopsy (EMB), Hysterosalpingogram (HSG), Endometrial biopsy, Sonohysterogram, Laparoscopy, Hysteroscopy, Thyroid tests, Chromosomes, Genetic Screening, Prolactin (PRL), hamster ovarian testing, hormonal assay (FSH, LH, prolactin, testosterone, progesterone, estrogen, DHEA-S)/ blood tests, Estradiol (E2), follicular ultrasound count, Clomid Challenge Test, PAP smear, abnormal PAP Smear, last mammogram, Semen Analysis, Allergies (Allergic to, Reaction), other
	**Previous Treatment**		Type and Date of Treatment, treatment outcome, antibiotics, clomiphene citrate with scheduled intercourse, clomiphene citrate with intrauterine insemination (IUI), clomiphene citrate with metformin, daily injection of IUI fertility, insulin sensitivity, Clomiphene citrate (Serophene, Clomid), hMG (Pergonal), danazol (Danocrine), Prednisone, Bromocriptine (Parlodel), Progesterone, Antibiotic, hCG (Profasi, Estrogens, urofollitropin or FSH (Metrodin), GnRH (Depo-Lupron), Daily fertility injections/gonadotropins (Gonal-F, Follistim, Menopur, Bravelle), Urofollitropin or FSH (Follistim, Metrodino), Gonal-Aff-Folium)/Hmg (O, Metrodino), / hCG (Profasi, Pregnyl)/ hCG(ProfessiONAL.P.LO), IVF, GIFT,ZIFT, other
	**Prior Surgeries**		Date, Procedure, Reason, type of surgery, surgery to open the fallopian tubes, surgery to remove adhesions, conjunctiva or cervical cavity, dilation and durettage (D&C) for abortion, for abortion after childbirth or for abnormal bleeding, any other surgery (ovarian, appendectomy, thyroid), bladder surgery during childhood, fallopian tube surgery, removal of the uterus, removal of the ovaries, surgery for sterilization/varicocele, bladder or penis surgery as child, vasectomy, vasectomy reversal, other
	**Infertility Treatment History (Prior IVF / ICSI cycles / or previous frozen embryos transfer)**		Dates, medications used and their total dosage, Peak estradiol, prior Clomiphene citrate (Serophene,Clomid) cycles, prior Letrozole (Femara) cycles, prior Gonadotropin cycles (Gonal-F, Follistim, Menopur, Repronex, Bravelle), Number of prior Fresh/ Frozen IVF cycles, Number of ovarian, adult ovarian, number of fertilized oocytes (2PN), ICSI, the number of embryos transferred, frozen embryos, embryos stages, outcome (pregnancy, delivery).
	**Disease History**		Height, weight, maximum weight, minimum weight of adulthood, unexplained weight loss, loss of more than 20 pounds in weight in the last year, excessive hair loss, eating disorders, Cryptorchidism, Cystic Fibrosis, cystitis, elevated Testosterone, Endocrine Problem, Endometriosis, Female Reproductive Problems, Galactoreaonorrhea, infertility factors, PCOS, tubal problems, Pelvic infection (PID), nipple discharge, Gonorrhea, Syphilis, Chlamydia, delayed puberty, diabetes, HIV/AHDS, Hepatitis, history of Mumps, difficulty with erection/ejaculation, retrograde ejaculation of sperm into the bladder, history of undescended testes, any serious genital injuries, any infection of the penis/testicle/prostate gland, hernia repair, contraceptive practices (IUD, birth control pills, tubal sterilization, injectable contraception), other
	**Medication History**		Drug, indication, dosage, Frequency, Duration, date, Anabolic steroids, Bravelle Estrogen Patch, Climara Estrogen Patch, Clomid (Serophene), Gonal F, Esrtodial, Follistim, Ganirelix, Lovenox, Pergonal, hCG Profasi, Repronex, herbal remedies or natural remedies, antibiotics, cortisone or steroids, Heparin, Antagon, Parlodel, baby Asprin, blood pressure pills, thyroid/ heart / urological drugs, sedative pills or nerves, Appetite suppressors or pep tablets, hormonal pills or shots, contraceptive practices (Birth control Pills), other.
	**Social History**		Alcohol/cocoa/caffeine drinking, cigarette smoking, recreational drugs usage (marijuana, heroin, cocaine), dietary supplements, herbal or sports supplements, exposure to (chemicals, X-rays, heat, steroids, cancer drugs, Lead, Poisons), exercise regularly, other.
	**Family History**		Any disease in the family, Cancer (Breast, Ovarian), infertility, Endometriosis, Cystic Fibrosis, fibroids, thyroid problems, infertility problems, history of recurring miscarriages, PCOS, pregnancy difficulties, anemia, autoimmune disorders such as lupus or rheumatoid arthritis, any birth defects, birth defects require surgery (cleft palate, lips), any inherited disorders, bleeding disorders (hemophilia, and others), other illness.
	**Pregnancy History**		Pregnancy length, total pregnancies, duration of pregnancy (weeks, miscarriage, full-term), number of full term deliveries, Method of Delivery (Vaginal, C-section), full-term (>37 weeks) birth, pre-term(<37 weeks) birth, biochemical pregnancies, the number of previous births (sex, weight), therapeutic (elective) abortions, spontaneous loss (miscarriages), number of ectopic/ tubal pregnancies, live birth, multiple birth, stillbirth, any pregnancies with birth defects, number of preterm deliveries (less than 37 weeks, preeclampsia/eclampsia, heavy bleeding, infertility treatment
	**Tests**	**Andrology Tests in men**	Semen analysis alone (sperm count, volume, motility, morphology), semen analysis and sperm DNA splitting test, repeat DNA division test (in 4 weeks after the initial test), semen analysis and ART appropriateness, IUI preparation for infertility into the uterus, donor preparation, sperm analysis with anti-sperm antibody testing, retrograde ejaculation analysis, spontaneous ejaculation analysis, semen/sperm freezing, post-vasectomy check, semen culture, FSH, Total Testosterone, Y microdeletion, Albumin, Prolactin, TSH, Free T4, Bioavailable Testosterone, Peripheral Blood karyotype, SHGB, Universal Plus, other.
		**Immunological examination in women**	Screening for Cystic Fibrosis, Rubella & Varicella (Chicken Pox) immunity. Clomiphene citrate (Clomid) challenge test (CCCT), Ovarian Assessment Report (OAR) through Reprosource, Progesterone (P4), Anti-phospholipid antibodies (APA or ACA), lupus anticoagulant (LAC), glycoprotein I Anti-ß2, anti-microsomal antibodies (AMA), antithyroglobulin antibodies (ATG), thyroid-stimulating hormone antibodies, HLA DQ, Glucose, DHEAS, 17 OHP, Prolactin, Testosterone, TSH, Free T4, LH, Hemoglobin A1C, Universal Plus, Fragile X, Uterine/Tubal Evaluation, HSG, SSG, other.
**Treatment plan**	**Features of the cycle**		Type of cycle associated with: oocytes, newly non-donated, newly donated, shared cycle, non-donated melting cycle, donation melting cycle, fresh cycle receptor, initiated cycles, Treatment code, cycle code, number of freeze-melted cycles in IVF and ICSI, start date of the cycle, other
	**specifications of eggs, Embryos / blastocyst profile, treatments**		Types of ART cycles or fertilization methods: in vitro fertilization (IVF), fertility treatment intracytoplasmic sperm injection (ICSI), gamete intrafallopian transfer (GIFT), frozen embryo transfer (FET), ovulation maturation, Pre-implantation genetic diagnosis (PGD), pre-implantation genetic screening (PGS), Surrogacy, with fresh ovulations, fresh donation of oocyte, new donation, embryo transfer, freeze transfer), IUI, IVF/ICSI, stimulant drugs: 1-GnRH agonist, GnRH antagonist, clomiphene citrate, hMG / FSH, LH, other, none, Ovum source: donated, non-donated, hot/ melted/warm, fresh donated, Sperm source: 1-partner/donation, 2-Non-donated ejaculation, 3- lack of non-donated ejaculation 4- Freezing, other.
**Treatment results**	**Treatment results: Pregnancy and childbirth**		Lack of follow up (pregnancy), transmission cycles, clinical pregnancy, clinical pregnancy per culture, missed pregnancy, completion date of pregnancy, live intrauterine pregnancy at week 7 or later, other.
	**Complicationss**		Complications (yes/no) 1- OHSS (high ovarian stimulation syndrome) 2. Pelvic infections requiring admission 3. Thrombosis 4. Bleeding 5. Complications of ovulation recovery 6. Other serious complications 7. Mother’s death, Associated medical disorders, chronic infections (hepatitis B, C, HIV), hospitalization due to complications, admission to assisted reproductive technology (ART), other.
**Donor information**	**Donor information**		First name, last name, date of birth, height, body mass index, and eye color, blood type, other.

Clinical data elements were categorized into four parts:

**Medical history:** Causes of Infertility, Sexual history (Menstrual history, Sexual history), Previous Examinations, Previous Diseases, Treatments, Tests, Surgeries, Medication, Social History, Family History, and Pregnancy History.

**Treatment plan:** Cycle Characteristics of the Treatment, Specifications of Eggs, Embryos/Blastocyst Profile, Treatments (Medication Treatments, Techniques Used in Treatment)

**Treatment results:** Pregnancy and Childbirth, Complications

**Donor information.**

## Discussion

Infertility affects over 15% of couples in reproductive age, and the prevalence of infertility in the world is increasing [13]. An accurate assessment of the prevalence and different etiologies of infertility is essential in order to plan appropriate strategies for the prevention, treatment, and management of health and socioeconomic outcomes [14]. Organizations need more comprehensive data collection and data planning in order to plan and, access to new and reliable information about the number of patients, diseases, methods and new therapeutic outcomes [10]. MDS is a tool with a variety of applications that can be implicated at the individual level in planning care and measuring outcomes and at the organizational level in quality management. MDS will facilitate comparison of data at the regional, national and international levels. The minimum data set can be dramatically used in the management of evidence-based healthcare [15].

In this study, based on the results obtained by using the Delphi technique, the majority of relevant experts agreed with the proposed model and it is considered suitable for infertility centers. Therefore, the proposed model as the final model of this research is recommended, and its implementation is recommended to infertility centers.

Various studies point to the importance of MDS in the development of information management system in different areas such as burns [9], trauma [16], biochemistry laboratories [17], antimicrobial resistance management (18), speech therapy (10), cystic fibrosis [19], C-section anesthesia [12], orthopedic injuries [20], organ transplantation [11], diabetes mellitus [21], aging [22], and echocardiography reporting system [23].

Identification of the minimum data set is the first step to standardize and integrate the data used in the diagnosis and can be useful in evaluating it [24]. MDS provides a comprehensive summary of the critical functional areas and the use of standardized definitions, and the response categories provide a common language in deciding on appropriate care plans and interventions that facilitate the assessment of multimedia teams and care planning. Due to reliability, MDS data can be used through the use of standardized information elements along with the same definitions and the provision of effective infertile management indicators for various purposes in addition to improving care [25]. Determining and identifying the minimum required data leads to better coordination in data collection and retrieval using information systems and allows us to identify and determine the required data [26].

## Conclusions

Considering the adverse effects of infertility, designing and implementing a comprehensive and appropriate infertility data set model in centers can be an essential step in the creation of infertility monitoring system for high-quality and proper treatment, regular planning for controlling and preventing infertility, allocating credit to infertility centers and clinics and doing research. MDS helps infertility management by providing continuous care of patients, establishing a link between care providers and analyzing the effectiveness of patient care and the community of infertile patients. Through the use of MDS organized as designated forms, patient identification indicators, efficiency indicators of the care process and the quality of the services provided as well as outcome indicators, fertility management was improved. In addition, it will be possible to develop policies, prevent and control infertility and, consequently, improve the quality of care and save costs.

It is clear that an important issue in preventing infertility is having access to credible and powerful information systems such as an infertility monitoring system. Although some countries develop and implement infertility management systems, data cannot be compared correctly due to differences in data and lack of international standards. Therefore, the provision of such standards will lead to the development of these systems in other countries.

## Acknowledgment

The authors would like to thank all the specialists from the Fasa University of Medical Sciences who participated or otherwise contributed to this study.

## Conflict of Interest

The authors confirm that there are no conflicts of interest.

## References

[1] Macaluso M, Wright-Schnapp TJ, Chandra A, Johnson R, Satterwhite CL, Pulver A (2010). A public health focus on infertility prevention, detection, and management. *Fertility and sterility*.

[2] Mascarenhas MN, Flaxman SR, Boerma T, Vanderpoel S, Stevens GA (2012). National, regional, and global trends in infertility prevalence since 1990: a systematic analysis of 277 health surveys. *PLoS Med*.

[3] Direkvand-Moghadam A, Sayehmiri K, Delpisheh A, Direkvand-Moghadam A (2014). The global trend of infertility: an original review and meta-analysis. *International Journal of Epidemiologic Research*.

[4] CDC (2014). A national public health action plan for the detection, prevention, and management of infertility. *Centers for Disease Control and Prevention, Atlanta, GA*.

[5] CDC (2013). Key statistics from the national survey of family growth.

[6] Hardee K, Gay J, Blanc AK (2012). Maternal morbidity: neglected dimension of safe motherhood in the developing world. *Global Public Health*.

[7] Malhotra N, Shah D, Pai R, Pai H, Bankar M (2013). Assisted reproductive technology in India: A 3 year retrospective data analysis. *Journal of human reproductive sciences*.

[8] Abdelhak M, Grostick S, Hanken MA (2014). Health Information-E-Book: Management of a Strategic Resource: Elsevier Health Sciences.

[9] Ahmadi M, Alipour J, Mohammadi A, Khorami F (2015). Development a minimum data set of the information management system for burns. *Burns*.

[10] Damanabi S, Abdolnejad S, Karimi G (2015). Suggested Minimum Data Set for Speech Therapy Centers Affiliated to Tabriz University of Medical Sciences. *Acta Informatica Medica*.

[11] Karimi S, Saghaeiannejad IS, Farzandipour M, Esmaeili GM (2011). Comparative study of minimum data sets of health information management of organ transplantation in selected countries and presenting appropriate solution for Iran.

[12] Sheykhotayefeh M, Safdari R, Ghazisaeedi M, Khademi SH, Farajolah SSS, Maserat E (2017). Development of a Minimum Data Set (MDS) for C-Section Anesthesia Information Management System (AIMS). *Anesthesiology and Pain Medicine*.

[13] Naser SSA, Alhabbash MI (2016). Male Infertility Expert system Diagnoses and Treatment. *American Journal of Innovative Research and Applied Sciences*.

[14] Safarinejad MR (2008). Infertility among couples in a population-based study in Iran: prevalence and associated risk factors. *International Journal of Andrology*.

[15] Poss J, Jutan N, Hirdes J, Fries B, Morris J, Teare G (2008). A review of evidence on the reliability and validity of Minimum Data Set data. *Healthcare Management Forum*.

[16] Ghodsi Z, Movaghar VR, Zafarghandi M, Saadat S, Mohammadzadeh M, Fazel M (2016). The Minimum Dataset and Inclusion Criteria for the National Trauma Registry of Iran: A Qualitative Study. *Archives of Trauma Research*.

[17] Shahmoradi M, Firoozabadi SM, Mohammadzadeh N (2016). The Local Minimum Dataset of Biochemistry Laboratory Information Systems.

[18] Safdari R, Saeedi MG, Masoumi-Asl H, Rezaei-Hachesu P, Mirnia K, Mohammadzadeh N (2018). National Minimum Data Set for Antimicrobial Resistance Management: Toward Global Surveillance System. *Iranian Journal of Medical Sciences*.

[19] Kalankesh LR, Dastgiri S, Rafeey M, Rasouli N, Vahedi L (2015). Minimum data set for cystic fibrosis registry: a case study in Iran. *Acta Informatica Medica*.

[20] Ahmadi M, Mohammadi A, Chraghbaigi R, Fathi T, Baghini MS (2014). Developing a minimum data set of the information management system for orthopedic injuries in iran. *Iranian Red Crescent Medical Journal*.

[21] Jahanbakhsh M, Moghaddasi H, Hosseini A (2010). Designing of diabetic mellitus minimum data sets: indicator basis of diabetic management effectiveness. *Health information management*.

[22] Kowal PR, Wolfson LJ, Dowd JE (2000). Creating a minimum data set on ageing in sub-Saharan Africa. *Southern African Journal of Gerontology*.

[23] Mahmoudvand Z, Kamkar M, Shahmoradi L, Nejad AF (2016). Determination of Minimum Data Set (MSD) in Echocardiography Reporting System to Exchange with Iran’s Electronic Health Record (EHR) System. *Acta Informatica Medica*.

[24] Bauer J, Sieber C (2006). Significance and diagnosis of malnutrition in the elderly. *Zeitschrift fur arztliche Fortbildung und Qualitatssicherung*.

[25] Hawes CH, Morris JN, Phillips CD, Fries BE, Murphy K, Mor V (1997). Development of the nursing home Resident Assessment Instrument in the USA. *Age and Ageing*.

[26] Avidan A, Weissman C (2012). Record completeness and data concordance in an anesthesia information management system using context-sensitive mandatory data-entry fields. *International Journal of Medical Informatics*.

